# Thyroid Surgery in Children: A 5-Year Retrospective Study at a Single Paediatric Surgical Center and Systematic Review

**DOI:** 10.3390/children9121818

**Published:** 2022-11-25

**Authors:** Svetlana Bukarica, Jelena Antić, Ivana Fratrić, Dragan Kravarušić, Miloš Pajić, Radoica Jokić

**Affiliations:** 1 Department of Paediatric Surgery, Institute for Healthcare of Children and Youth of Vojvodina, 21000 Novi Sad, Serbia; 2 Department of Surgery, Faculty of Medicine, University of Novi Sad, 21000 Novi Sad, Serbia

**Keywords:** thyroid gland, children, thyroid nodule, papillary carcinoma, thyroid surgery

## Abstract

The aim of this study was to analyse and evaluate our 5-year experience in paediatric thyroid surgery, as well as the specificities of this kind of surgery in the literature. This retrospective study was based on 19 operations in 17 patients aged from 5 to 17 years who were operated on due to thyroid pathology from 2017 until 2022. We presented data on surgical procedures and complications following surgery. Most of the patients were adolescent girls. The most common clinical presentations included enlarged thyroid gland, followed by thyroid nodules and hyperthyroidism. Eight total thyroidectomies, five left lobectomies, five right lobectomies, and three central neck dissections were performed. The most common histopathological diagnosis was hyperplastic diffuse colloid goitre, followed by papillary carcinoma, cystic nodule, follicular adenoma, Hashimoto thyroiditis and toxic adenoma. Postoperative course was uneventful, with four mild complications (one wound infection, one manifest hypocalcaemia, and two transitory recurrent laryngeal nerve paralysis). In our literature review, eighteen full-text articles were included and analysed. This study demonstrated that thyroid surgery in paediatric population is a safe and efficient procedure. Thyroid pathology in children significantly differs from that in the adults, and paediatric surgeons should be included into the team managing such cases.

## 1. Introduction

Thyroid pathology is uncommon in children. However, hypothyroidism, hyperthyroidism, thyroid nodules and thyroid carcinoma are all reported in the paediatric population [1].

The incidence of hyperthyroidism is 0.8/100,000 for children younger than 15 years of age [2]. Up to 30% of them will need some surgical intervention due to lack of remission, side effects of the prescribed drugs, an extremely large goitre that cannot be treated with radioactive iodine, or toxic adenoma [2]. A palpable nodule or nodule that is only detected on ultrasonography requires an analysis of TSH levels. If TSH is suppressed, scintigraphy is obligatory. Hyperfunction of the nodule should be referred for surgery. If TSH is not suppressed, fine-needle aspiration biopsy (FNAB) is indicated. Malignant cells detected by FNAB, or even suspicion of malignancy, require surgery for the final histopathological evaluation [3].

One percent of all thyroid cancers affect children aged 5–9 and up to 7% of adolescents (from 15 to 19 years) [4,5]. Firm, non-tender nodules in the thyroid or enlarged lymph node in the neck region should prompt suspicion of thyroid cancer, especially if associated with rapid growth, hoarseness and lung metastases. The incidence of malignancy is from two to three times higher in children than in the adult population [4,5]. The most common type of thyroid malignancy in children is papillary carcinoma, followed by follicular carcinoma, while anaplastic and medullary carcinoma are exceedingly rare in the paediatric population. Almost all medullary carcinomas in the paediatric population are hereditary and part of Multiple Endocrine Neoplasia Type 2 (MEN 2B).

The guidelines of the American Thyroid Association for the paediatric population highlight the necessity for preoperative ultrasonography and FNAB [3]. The preoperative diagnosis of malignancy requires radical surgical operation in the form of total thyroidectomy with occasional central neck dissection (involvement of lymph nodes). Radioactive iodine is reserved for cases of persisting forms of the disease, as it could lead to secondary malignancy [3]. The standard follow-up of paediatric patients with thyroid cancers includes ultrasonography and detection of thyroglobulin blood levels [3].

The well-known Bethesda system classifies thyroid fine-needle aspiration cytology (FNAC) into six categories (I—nondiagnostic or unsatisfactory, II—benign cytopathology, III—atypia of undetermined significance or follicular lesion of undetermined significance, IV—follicular neoplasm or suspicious for follicular neoplasm, V—suspicion of malignancy, VI—malignant). Monteiro et al., 2018 highlighted that the risk of malignancy in Bethesda III category in children is up to 30%, while this risk rises to 60% in Bethesda IV and up to 100% for children with Bethesda V [6].

The only test that can detect increased risk of malignancy is molecular testing (BRAF^V600E^, RET/PTC gene and NTRK–fusion) [7]. In the case of inconclusive molecular testing or if those tests are unavailable, diagnostic lobectomy is an appropriate surgical option. If mutations on these genes are detected, there is a 100% risk of malignancy and total thyroidectomy should be performed.

Surgical options in the treatment of paediatric thyroid pathology include: total thyroidectomy, near-total thyroidectomy, subtotal thyroidectomy or lobectomy [8]. Total thyroidectomy comprises extracapsular extraction of the thyroid gland, while in near-total thyroidectomy, a capsule is entered and a small amount of thyroid tissue is left near the parathyroid gland and recurrent laryngeal nerve to avoid damage. Subtotal thyroidectomy consists of the removal of one lobe of the thyroid, the isthmus, the medial portion of the other thyroid lobe. Finally, lobectomy refers to the resection of anatomic lobe and isthmus. Additionally, lymph node surgery may be added to thyroid surgery. The extraction of only affected lymph nodes, so-called “berry picking” surgery, is rarely used at present. However, compartmental lymph node dissection, when all lymph nodes of one compartment are removed, is used more often. Central lymph node dissection is the most common lymph node operation, while lateral and mediastinal compartments are rarely affected [8]. Radioactive iodine therapy in paediatric population is reserved for progressive forms of disease, several months after the primary surgical procedure [8,9].

The aim of this study was to analyse and evaluate our 5-year experience in paediatric thyroid surgery, as well as the specificities of this kind of surgery, in the literature.

## 2. Materials and Methods

Medical data from 17 patients operated at the Clinic for Paediatric Surgery at the Institute for Healthcare of Children and Youth of Vojvodina were collected and analysed.

This study was designed as a retrospective observational study. Medical data such as age, gender, preoperative diagnosis and treatment, type of thyroid surgery, histological analysis, postoperative complications, and duration of hospitalization were recorded for each patient.

The primary expected outcome of the study was to analyse the demographical characteristics, type of thyroid surgery, histological analysis and postoperative complications in patients undergoing an operation on the thyroid gland at our clinic.

The secondary expected outcome of this study was to perform a systematic literature search in the field of thyroid surgery in children.

All patients operated at our clinic during the study period were included in the study.

**Figure 1:**
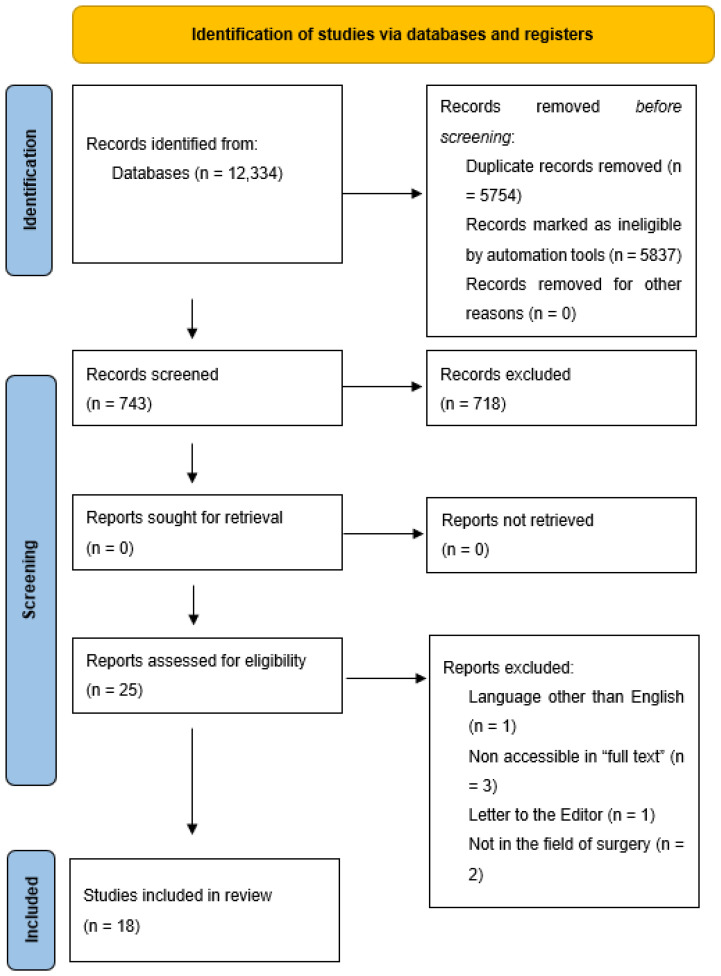
PRISMA 2020 flow-diagram for new systematic reviews which included searches of databases.

A systematic review was performed as per the Preferred Reporting Items for Systematic Reviews and Meta-Analyses (PRISMA) guidelines [10]. Two investigators (R.J.; I.F.) independently searched PubMed, Excerpta Medica database (EMBASE), Web of Science, and Scopus databases on 3 November 2022 (Figure 1)**.** The search keywords were ((thyroid surgery) OR/AND (children)). The total search records were identified, and duplications were removed. After limiting the date of publishing to the last five years, we excluded articles that were not written in English, those that were not available in “full text”, letters to the editor and other meta-analyses, and limited our results to the surgical aspects of thyroid disease. Finally, 18 full-text articles were included in our review.

### 2.1. Operative Technique

**Figure 2:**
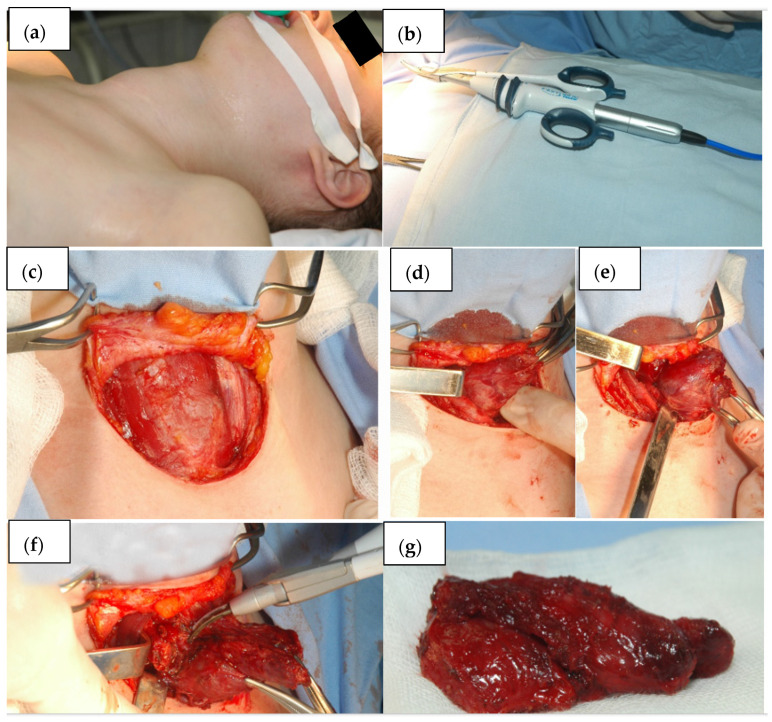
Thyroid lobectomy with isthmusectomy. (**a**)—Patient before the operation, (**b**)—ultrasonic surgical device, (**c**)—upper and lower skin flap is formed under the platysma, (**d**,**e**)—mobilisation of the thyroid gland, (**f**)—ligation and resection of the thyroid artery, (**g**)—thyroid lobe with isthmus.

**Figure 3:**
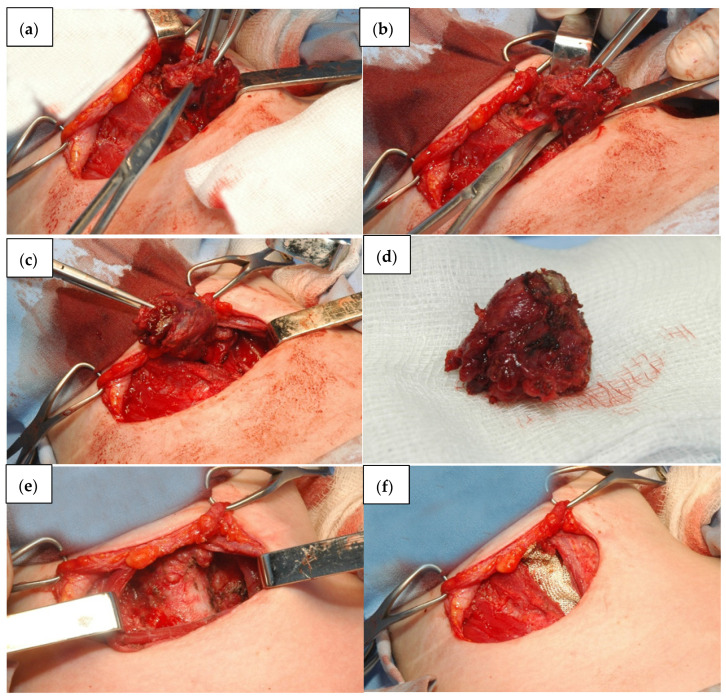
Steps of total thyroidectomy in pediatric patient. (**a**)—left thyroid lobe is approached, (**b**,**c**)—mobilization of the left lobe of the thyroid gland, (**d**)—left thyroid lobe, (**e**)—the appearance of the trachea after total thyroidectomy, (**f**)—absorbable haemostat left on site for bleeding control.

During this surgical procedure, the patient is usually in supine position with the head extended. Surgery starts with the collar symmetric incision on the base of the neck, about 2 cm above the jugulum. Upper and lower skin flaps are formed under the platysma. Avascular white line of the neck is then incised, and visceral region of the neck is entered between infrahyoid, sternothyroid and sternohyoid muscles. Mobilization of the thyroid gland is started from the lateral part, where the medial thyroid vein is detected, ligated and resected. Then, parathyroid glands and laryngeal recurrent nerve are identified and preserved. The next step is the identification of the upper and lower thyroid artery, their ligation and resection. Isthmus is then transected and that completes lobectomy (Figure 2). Total thyroidectomy involves the removal of the entire thyroid gland (Figure 3). Bleeding is controlled by compression, thermo cauterization, ultrasonic surgical devices, ligation of blood vessels and placement of absorbable haemostats (Surgicel ^R^). Most often, drainage is also carried out.

### 2.2. Ethical Statement

This retrospective study was approved by the Ethical Committee of the Institute for Child and Youth Healthcare of Vojvodina, Serbia (code 2968-1 from 1 July 2022).

### 2.3. Statistical Analysis

Medical data were analysed using SPSS Statistics 20.0 (IBM Corporation, Armonk, NY, USA) and Microsoft Excel 2010 program (Microsoft Corporation, Redmond, WA, USA). Descriptive parameters were used in the analysis.

## 3. Results

We performed 19 thyroid operations in 17 patients in the 5-year period January 2017–March 2022.

There were 14 girls and 3 boys operated for thyroid pathology. Female predomination is noted, with a female to male ratio of 4.7:1.

Majority of children had no symptoms before the surgery. Others reported difficulties in swallowing (5 patients), neck tightness (2 patients), heart palpitations (1 patient), weight loss (1 patient) and cold extremities (1 patient).

Table 1 shows the number of operations throughout the observed period. An increasing number of operations was noted, even during COVID-19 pandemic, despite the restrictions in the number of planned procedures.

The incidence of thyroid malignancy increased in adolescent years (3 patients aged from 16 to 18 years and one in the category from 10 to 16 years). Only one patient younger than 10 years was operated, due to a benign nodule in the thyroid gland.

Additionally, an increased number of paediatric patients with thyroid cancer was recorded in our study in the last 5 years compared with the previous 19 years (from 1998 to 2017), when we operated only two paediatric patients with thyroid malignancy.

We performed 10 lobectomies and 8 total thyroidectomies. Correlations with histopathological diagnosis are shown in Table 2.

Patients were hospitalized for from 2 to 8 days. Median hospital stay was 4 days.

During the study period, we had only several mild complications: one wound infection that resolved well on antibiotic therapy and wound dressings (Clavien Dindo classification II [11]), one manifest transitory hypocalcemia treated with short-term substitutional therapy (Clavien Dindo classification II [11]), and two temporary laryngeal nerve paralyses that resolved well on neurotropic medicines, glucocorticoids and vasodilators (Clavien Dindo classification II [11]).

A literature review of eighteen articles dealing with thyroid surgery in paediatric population is shown in Table 3 [12,13,14,15,16,17,18,19,20,21,22,23,24,25,26,27,28,29]. Most studies (18) included in our literature review were retrospective studies. Three case series were also included in our review. Study period varied from 1 year [17] to 18 years [22]. Studies with the most patients were those in which partial or total thyroidectomy was usually performed [12,13,15,18,19,20,21,22,23,24,25,26,27,28,29], while fewer patients were included in studies that analysed robotic trans-axillary and retro-auricular approach [14] or transoral endoscopic thyroidectomy via vestibular approach [17]. The most common complication in all operations was hypocalcaemia due to incidental parathyroidectomy and recurrent laryngeal nerve injury. Hematoma, chyle leak, lymph fistula, surgical site infection, temporary shoulder hypoesthesia, oculo-sympathetic paresis and recurrence of thyroid carcinoma were also reported.

## 4. Discussion

Our previous education in this field included direct cooperation with adult endocrine surgeons; however, an independent paediatric surgical team for thyroid pathology was created in 1998 at our Institute. In this way, the concept of managing childhood pathology by paediatric surgeons is supported. Although it is hard for the paediatric surgeon to attain a high number of performed operations due to the low incidence of thyroid pathology in children, it is of great importance to maintain as many operations as possible in order to lower the number of complications. Many authors [12,30] are still debating as to who should be operating on children with thyroid pathology. At present, we are the only children’s surgery centre in our country that independently manages thyroid pathology and we are among the rare children’s surgery centres that have experience in this field.

Female predominance in thyroid pathology is well known and reported in the literature [3,16,18]. Our study shows a similar result, with a female to male ratio of almost 5 to 1.

The symptoms of thyroid gland dysfunction may be lacking at the beginning, and thyroid pathology is suspected and diagnosed as late as the enlargement of thyroid gland or nodule. In our study, the majority of children were without any symptoms. The symptoms included swallowing difficulties and neck tightness, probably because of thyroid enlargement. Other symptoms, such as heart palpitations and weight loss, were probably due to hyperthyroidism. Unlike these manifestations, cold extremities reported in one child could be the consequence of hypothyroidism.

We noticed an increasing number of patients operated due to thyroid pathology in recent years. The literature review also shows an increasing number of operated patients in the recent years, which could be explained by the higher overall incidence of thyroid pathology [3,21]. We assume that the number of performed operations would be even higher if the COVID-19 pandemic did not occur. This pandemic has delayed a number of planned operations. However, infection with SARS-CoV-2 has been shown to affect thyroid function, which accelerated some pathologic conditions in the thyroid gland [30].

Adolescent girls are at higher risk of thyroid carcinoma, as also reported in our study. The study from 2022, conducted by Moreno Alfonso et al. [21], showed 66% malignancy in children operated for thyroid nodule. However, 26% of thyroid nodules were found to be malignant in the study of Divarce et al. [19]. In almost one quarter of our patients (23.5%), histopathological analyses revealed malignancy, which is up to 45% of children operated for thyroid nodule at our clinic.

In all cases of thyroid malignancy that we reported here, papillary carcinoma was detected. No follicular, anaplastic or medullary carcinomas were found. However, in the period from 1998 until 2017, we operated on one child with medullary carcinoma, which implies that even a greater diapason of malignant thyroid pathology was operated at our center then shown in this study. Children with papillary carcinoma have better overall survival compared with other forms of thyroid cancers, and it is well-known that papillary carcinoma is the most common thyroid malignancy in the paediatric population [5].

Although uncommon in paediatric population, thyroid cancer in children shows a higher probability of presenting as a bilateral and multifocal disease in comparison to the adult population. For this reason, indications for total thyroidectomy in children differs from that in adults, and is more often obligatory. The American Thyroid Association guidelines for management thyroid nodules and differentiated thyroid cancer in children [3] state, in recommendation 11: “For the majority of children, total thyroidectomy is recommended. The rationale for this approach is based on multiple studies showing an increased incidence of bilateral and multifocal disease. In long-term analysis, bilateral lobal resection compared with lobectomy has been shown to decrease the risk for persistent/recurrent disease”. The same association, in its guidelines for adult patients [31], in Recommendation 35, states: “For patients with thyroid cancer >1 cm and <4 cm without extrathyroidal extension, and without clinical evidence of any lymph node metastases (cN0), the initial surgical procedure can be either a bilateral procedure (near-total or total thyroidectomy) or a unilateral procedure (lobectomy). Thyroid lobectomy alone may be sufficient initial treatment for low-risk papillary and follicular carcinomas; however, the treatment team may choose total thyroidectomy to enable radioactive iodine (RAI) therapy or to enhance follow-up based upon disease features and/or patient preferences”. Following the guidelines for children, all patients with thyroid cancer in our study were treated with total thyroidectomy. An example of proper differences in the treatment of adults and children with thyroid pathology is shown in one of our patients in whom lobectomy was performed initially. After a histopathological analysis of thyroid cancer in the resected lobe, total thyroidectomy was performed as a second operation, despite the suggestion of experienced adult endocrine surgeons. Undoubtedly, cancer cells were detected in the other lobe of the thyroid gland. This also confirms the fact that thyroid cancer in children is usually a bilateral and multifocal disease, and indications for total thyroidectomy are different than those in adults.

According to the most recent guidelines from American Thyroid Association, thyroid operations are advised to be performed by a high-volume surgeon in the paediatric population [3]. A high-volume surgeon is usually defined as surgeon that performs at least 30 operations per year. This might be the reason that these operations are rarely performed by paediatric surgeons. However, Drew et al. [12], in their study on 3149 paediatric patients, emphasized the rarity of thyroid pathology in children, and defined a “high-volume” surgeon as surgeon who performs at least nine thyroid operations per year [12]. Our study mainly investigated the period of the COVID19 pandemic, which could be the reason for the small sample size. Other paediatric surgical centers are also in a dilemma regarding who should be operating on our children when discussing thyroid surgery [30]. As the number of the thyroid operations is increasing, there is a great probability that the goal of nine operations per year will be achieved in the forthcoming period, which is necessary for the definition of a “high-volume” surgeon in the paediatric population [12].

Unlike previous recommendations, when so-called “berry picking” of lymph glands was performed and only suspected lymph nodes were evacuated and sent for histopathological analysis, the most recent guidelines refer to central neck dissection whenever there is confirmed lymph node involvement [3]. Recent findings suggest that profilactic central neck dissection increases disease-free survival in pediatric papillary carcinoma without increase in postoperative complications [32]. In our study, we performed three central neck dissections in patients with papillary carcinomas.

The few complications that were reported are in accordance with the literature findings [3,13,16,18,19,23,24,25,26,27,28,29]. Infection of the wound is well-known and possible complications occur after every surgical procedure. In our case, this was resolved with antibiotic treatment and regular dressings. Manifest hypocalcemia is one of the most frequent postoperative complications after thyroid surgery [13,29]. Transient, mild hypocalcemia is reported in from 15% to 30% of patients [10]. Manifest severe hypocalcemia is less common. This is reported in from 1% to 7% of patients after thyroid surgery [10]. It is well-known that hypocalcemia is the result of extraction or vascular damage to parathyroid glands [13]. Recent findings reveal that near-infrared fluorescent imaging could help visualize parathyroid glands, and thus decrease the rate of the parathyroid damage and consequently patient’s hypocalcemia [10,33,34]. Another recent finding showed that indocyanine green could be used during thyroid surgery for the same reason [35].

Another important complication after thyroid surgery is recurrent nerve palsy. Vocal cord paresis or paralysis that is the result of the injury of recurrent laryngeal nerve represents major postoperative disability. The treatment for recurrent nerve palsy includes voice therapy, early reinnervation techniques, vocal fold medialization techniques that include medialization thyroplasties, injection laryngoplasty, arytenoid adduction and laryngeal reinnervation [36]. In order to lower the rate of this complication, many centres are now using intraoperative neuromonitoring as an addition to visual nerve identification during operation [37]. It has been two decades since the introduction of neurostimulation of the recurrent laryngeal nerve during thyroid surgery of children and adolescents [38], and new modifications with the transcartilage recording method are already available [39], instead of using conventional endotracheal tube electrodes. Despite the innovations in the neuromonitoring of the recurrent laryngeal nerve, this is still not widely used in the paediatric population.

A recent literature review revealed a small number of published articles focused on paediatric thyroid pathology [12,13,14,15,16,17,18,19,20,21,22,23,24], especially those produced by paediatric surgeons on their own (singly) [12,20,21,24] or in cooperation with adult endocrine surgeons [18,25]. In the majority of articles, total thyroidectomy was the dominant surgical procedure, in accordance with the American Thyroid Association and European Thyroid Association guidelines.

Novel paediatric surgery complies with minimally invasive principles, which is reflected in the implementation of robotic surgery with innovative approaches, such as the transaxillary [14,15] or retroauricular approach [14]. The transoral endoscopic approach through oral vestibulum is also introduced in clinical practice [17]; however, there are scarce data available on this procedure, and the applicability assessment requires a higher number of cases and randomized trials.

Although the multidisciplinary team should include an endocrine surgeon, paediatric surgeon and ORL, who may be the most experienced in the treatment of thyroid surgery, a maxillofacial surgeon, endocrinologist, radiologist, oncologist and pathologist are all an important part of the team to obtain the best treatment results. What we suggest is higher experience and education for paediatric surgeons in this field, as we identify the paediatric surgeon as potentially being the leader of this multidisciplinary team. The most important and still-valid requirement for surgeons performing this kind of surgery is the number of completed operations, as this lowers the rate of postoperative complications in this very sensitive paediatric population.

The limitations of our study include the small sample size, possible impact of surgeon experience in reported outcomes, procedural details, which may vary, and the single-center design of the study.

## 5. Conclusions

Thyroid surgery in children is a safe and efficient procedure. The most frequent procedure in children is total thyroidectomy. A multidisciplinary approach is essential and includes an endocrine adult and paediatric surgeon, maxillofacial surgeon, otorhinolaryngologist, endocrinologist, radiologist, oncologist and pathologist. The existing differences in the treatment of paediatric patients with thyroid pathology, in comparison to th eadult population, require more education and clinical practice for paediatric surgeons, who are potential leaders of this multidisciplinary team.

## Figures and Tables

**Table 1:** Number of operations through the years.

Year	Number of Operations	Mean Age of the Patient (Years)
2017	5	15.6
2018	2	14.5
2019	1	16.0
2020	3	14.3
2021	6	11.5
2022	2	17.0
Total	19	14.8

**Table 2:** Pathohistological diagnosis in patients with different type of surgical procedure.

Surgical Procedure	Number of Patients	Pathohistological Analysis
Total thyroidectomy	8	Struma, Graves’ disease, papillary carcinoma
Lobectomy	10	Benign nodules, Hashimoto thyroiditis, struma
Central neck dissection	3	Papillary carcinoma

**Table 3:** Review of the literature findings.

Study	Study Period	Study Design	Patients	Performed Surgery	Surgeon	Complications
Drews et al. [12]	2005–2016	Retrospective cohort study	3149	Partial or total thyroidectomy	High- vs. low-volume surgeon, pediatric surgeon vs. ORL	hypocalcemia, recurrent laryngeal nerve injury, hematoma, chyle leak, and surgical site infection
Sahyouni et al. [13]	2010–2020	Retrospective cohort study	209	Lobectomy or total thyroidectomy, with or without neck dissection	No data	Incidental parathyroidectomy
Wu et al. [14]	2010–2017	Case series	7 (9 surgeries)	Robotic transaxillary and retroauricular approach	high-volume surgeon skilled in robot-assisted operations for neck diseases in adults	temporary shoulder hypoesthesia, temporary vocal cord paresis, transient symptomatic hypocalcemia
Lee et al. [15]	2008–2019	Retrospective study	161	transaxillary (99) or conventional open thyroidectomy (COT) (62) for thyroid cancer	Six surgeons were experts on robotic thyroidectomy and two doctors performed only COT	transient or permanent hypocalcemia, chyle leak, wound infection, transient or permanent RLN injury, and oculo-sympathetic paresis
Almosallam et al. [16]	2000–2014	Retrospective study	103	Thyroidectomy	No data	hypocalcemia, unilateral recurrent laryngeal nerve injury
Ngo et al. [17]	2020	Case series	4	Transoral endoscopic thyroidectomy via vestibular approach	high-volume thyroid surgeon	No
Van Rooijen et al. [18]	2013–2020	Retrospective single center study	48	29 total and 19 hemithyroidectomies	Surgeon and endocrine surgeon (>50/year)	Rapidly resolved hypocalcemia, transient hypocalcemia and permanent hypocalcemia
Divarci et al. [19]	2006–2014	Retrospective study	58	nodulectomy/lobectomy (32), total thyroidectomy (TT) (13), or TT + neck dissection (13)	No data	Postoperative complications were transient hypocalcemia, permanent hypocalcemia and unilateral vocal cord paralysis
Merchant et al. [20]	No data	Case series	3	Total thyroidectomy for McCune Albright syndrome at an early age	Pediatric surgeon	No
Moreno Alfonso et al. [21]	2010–2020	Retrospective study	11	thyroidectomy ± cervical lymphadenectomy	Low-volume pediatric surgeons	transient hypocalcemia, transient recurrent laryngeal nerve neuropraxia
Gui et al. [22]	2000–2018	Retrospective study	95	Thyroidectomy for papillary thyroid carcinoma	High-volume surgeon	30% of recurrence
Staubitz et al. [23]	2005–2018	Retrospective single center study	155	Total thyroidectomy +/− lymph node dissection, subtotal, partial thyroidectomy, lobectomy	Endocrine surgeon	Transient hypoparathyroidism, permanent hypoparathyroidism, wound infections, transient vocal cord palsy, lymph fistula
Spinelli et al. [24]	2000–2017	Retrospective multicenter study	30	Lobectomy, total thyroidectomy +/− neck dissection	Pediatric surgeon	Transitory hypoparathyroidism, definitive hypoparathyroidism, lesion of the recurrent laryngeal nerve
Elgendy et al. [25]	2011–2021	Retrospective sudy	32	Hemithyroidectomy or total thyroidectomy +/− neck dissection	Team (oncology surgeon, ORL, pediatric surgeon)	Chyle leak, hypoparathyroidism, nerve palsy, relaps
Chen et al. [26]	2006–2015	Retrospective study	276	Lobectomy or total thyroidectomy +/− neck dissection	High-volume thyroid surgeons	Recurrence
Sudoko et al. [27]	2009–2020	Retrospective cohort study	102	Lobectomy or total thyroidectomy +/− neck dissection	No data	No data
Spinelli et al. [28]	2014–2019	Retrospective study	76	Thyroidectomy hemithyroidectomy, lymphadenectomy	No data	Recurrent laryngeal nerve palsy, transient hypocalcemia, permanent hypocalcemia
Nordenstrom et al. [29]	2004–2014	Retrospective (population-based)	274	Total thyroidectomy	No data	Permanent hypoparathyroidism

## Data Availability

All source data included in this paper are available at the Institute for Child and Youth Healthcare of Vojvodina.
